# Using Stakeholder Involvement, Expert Knowledge and Naturalistic Implementation to Co-Design a Complex Intervention to Support Children’s Inclusion and Participation in Schools: The CIRCLE Framework

**DOI:** 10.3390/children8030217

**Published:** 2021-03-11

**Authors:** Donald Maciver, Cathleen Hunter, Lorna Johnston, Kirsty Forsyth

**Affiliations:** 1 School of Health Sciences, Queen Margaret University, Edinburgh EH21 6UU, UK; chunter@qmu.ac.uk (C.H.); LJohnston2@qmu.ac.uk (L.J.); kforsyth@qmu.ac.uk (K.F.); 2 Additional Support for Learning Service, Communities and Families, City of Edinburgh Council, Edinburgh EH8 8BG, UK

**Keywords:** inclusive education, complex interventions development, teachers, health professionals, qualitative, co-production, co-design

## Abstract

Whist inclusion is recommended for most children most of the time it remains difficult to implement. In this paper, we present the process undertaken to review and redesign a pre-existing complex intervention (The CIRCLE Framework) which was designed to enhance teachers confidence and competence in provision of universal first level supports for 5–12 year old children with additional support needs. The approach presented draws on the Medical Research Council guidance for the development of complex interventions. A series of ten co-design workshops with 70 stakeholders was completed, applying interactive and participatory methods. Analysing outputs of each workshop revealed recurring design ideas that became the main aspects of the new framework and associated manuals. Intervention content, theoretical frameworks, manuals to support use in practice and implementation strategies were developed. On completion, the updated intervention was extended up to 18 years of age and redistributed to all teachers in the participating local authority. We present the main conclusions and interpretations around the design and naturalistic implementation of the framework, and reflections on use in practice, including a detailed list of recommendations for implementation across schools and staff.

## 1. Introduction

Increasingly, children with additional support needs are educated alongside peers in inclusive schools [1,2,3,4,5]. There are numerous studies and theoretical perspectives associated with what makes an effective inclusive school [6]. Core principles that should underpin comprehensive school reform to facilitate inclusion are widely accessible. However, teachers still have difficulty operationalising these concepts, and including learners with additional needs. There are issues with attitudes to inclusion and disability [7], understanding of specific needs, for example autism and other neurodevelopmental disorders [8], and level of knowledge of staff [9], leading to ongoing calls for more training, support, and resources [10]. Attempting to reduce unequal outcomes for all children is the primary goal [11]. Within this context, we developed a novel intervention to improve the confidence and competence of teachers around inclusion and participation in the school setting [12]. This paper presents an analysis of the steps taken to improve and extend this intervention.

Modern methods of intervention development, for example the Medical Research Council complex interventions development framework [13], are systematic and theory-driven approaches to developing novel interventions. Developing the evidence base for new interventions may include a range of methods. Taking an approach based on quantitative statistical techniques, such as meta-analysis or controlled trial, whilst desirable, is not possible when robust quantitative data does not exist for the intervention or population [14]. Qualitative research is a useful alternative [15,16]. Qualitative methodologies can be used to develop new understanding about how to deliver interventions, mechanisms and outcomes of interventions, and key features of interventions believed to be important [17]. Extensive stakeholder involvement is of fundamental importance, helping to identify priorities, understand problems and find solutions that will work in the real world, ensuring that interventions are realistic and effective for their context [18].

This paper presents an intervention development process, drawing on techniques of qualitative research and stakeholder involvement. The process covers the review and redevelopment of the CIRCLE Framework, an intervention that was designed to support teachers to be inclusive practitioners and to work effectively with children with additional support needs in schools.

### 1.1. Background and Context

In Scotland, where this research was carried out, “additional support needs” is the term used to describe children who require support over and above that which is typically required. Direct comparison across countries are complicated by differing systems and definitions, however, analogous terms internationally are Special Educational Needs (SEN) and/or Special Educational Needs and Disabilities (SEND). In line with international trends, numbers of children with additional support needs in the school system have been rising significantly in Scotland. In 2012, 16.9% of 5–12 year old children (62,572 children) were recorded as having one or more additional support need, rising to 27% in 2019 (107,635 children) [19]. For secondary schools (12–18 year old learners), 16.5% of learners were recorded as having one or more additional support need in 2012 (48,486 learners) compared to 35% in 2019 (101,130 learners) [19]. Additional support needs are commonly identified for autistic children, children with social and emotional needs, children with learning difficulties and children with speech or language disorders [19] [for more information, including a breakdown of neurodevelopmental disorders, see Supplemental Materials A1–A4]. Segregated specialist schools are available, but this is for a very small minority. In 2019 there were 7132 children registered for special education in Scotland (across primary and secondary education) [19]. Therefore, it is clear that the focus on inclusion has led to the majority of children being educated in general education, leading to a subsequent interest in tools and methods to improve inclusion for this group [20].

Placement is no longer the key determinant, i.e., special vs. other schools [21]. From a rights-based perspective, inclusion is an ethical priority [22]. Contemporary practice focusses on ensuring children’s participation and inclusion through changes to the school, with particular emphasis on staff behaviours, environments, routines and structures. Most commentators broadly support the case for inclusion, indicating that there is a strong argument, and some empirical evidence, that inclusive educational settings confer benefits [23,24]. However, the strength of the evidence is variable [25] and hampered by lack of clear definitions and theoretical frameworks [26]. A recent review has identified that the field has a large preponderance of theory and policy; however, research that actually develops, applies, and adds evidence on how support should be provided is less common [27]. A key issue is an overt focus on individual conditions leading to a “program for every problem” [28]. An overabundance of programs makes getting it right for children with additional needs difficult. There are also rights-based issues around making sure children are not reduced to their limitations [21]. Research indicates that teachers often require new skills and knowledge to work with diverse learners, leading to recommendations that additional training around inclusive education is necessary within initial teacher training and continuing professional development [29].

Considering the above, a cross-discipline partnership was developed between education staff, health professionals and academics to address these issues. The title of the partnership was the Child Inclusion: Research into the Curriculum, Learning and Education (CIRCLE) Collaboration. This group undertook the development of the CIRCLE framework [12]. This framework was in response to a requirement for a classroom teacher focussed intervention for use in mainstream schools to improve teachers’ practices.

### 1.2. Initial CIRCLE Development and Evaluation

The first version of the CIRCLE framework was developed using a literature review and a 2-year period of qualitative research [12]. The qualitative studies involved in-depth interviews and observation of professionals from education (working in mainstream and specialist settings), health staff and parents/carers of children aged 5–12 years [12]. The CIRCLE development team included Head (Principle) Teachers, Educational Psychologists, a range of specialists in inclusive practice and additional support for learning, and senior paediatric specialists from occupational therapy, speech and language therapy, and physiotherapy, in addition to academics and clinical researchers. The development team helped to synthesise key practices identified from analysis of the preceding research. Through a cyclical process of review and refinement, this led to the development of the CIRCLE framework and a manual that was considered reflective of best practice.

The CIRCLE framework and associated manual were designed to provide a universal first level framework for teachers and other related staff. Reflecting the social model of disability, the CIRCLE framework emphasises embracing difference and the importance of considering and adapting environmental factors. As such, the framework and manual include strategies based on this set of ideas to support adaptations to structures and routines, modifications to the physical environment and teacher approaches for individual children. A key underpinning concept is the premise that supporting children of all abilities is not the remit of specialists, but the duty of everybody; and that all teachers within the school can and should provide support for all children.

Initial research found that the CIRCLE framework was received well [12]. Teachers and other school staff reported it was a useful resource [12]. Teachers reported that the framework and associated manual were feasible and acceptable, and supported them to be systematic in their approach to meeting the needs of children with additional needs [12]. Users reported that the framework and manual were useful in supporting joint working by providing a common language for collaboration [12]. Following the initial research, CIRCLE was disseminated across the local education authority, which was an urbanised area encompassing c85 primary schools, c1400 teachers, c30,000 primary aged children.

In view of its perceived utility, the local authority commissioned further development of the CIRCLE framework and expanded the remit to include support for older children (12–18 years). This process is covered in this paper.

## 2. Materials and Methods

### 2.1. Ethical Approval

Ethical approval was provided by University Research Ethics committee (Queen Margaret University “CIRCLE Project” 01062013) and permission to work with teachers and schools was granted by the Local Authority Research Access Service. Written informed consent was obtained from workshop participants and anyone who provided qualitative data. All participants were volunteers and given the opportunity to withdraw at any time without giving a reason.

### 2.2. Process in Development of the Updated CIRCLE Framework

**Figure 1:**
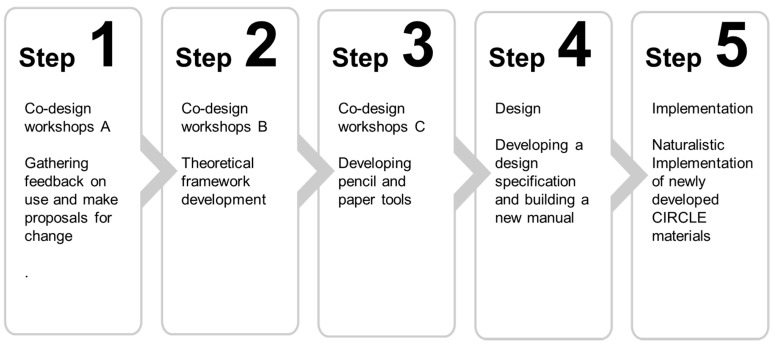
Process for development of CIRCLE framework.

Following the Medical Research Council complex interventions development framework [13] several steps were completed, encompassing co-design workshops, stakeholder involvement, theory development, and naturalistic implementation. See Figure 1. This paper presents each of these stages in turn, covering methods and outputs sequentially.

## 3. Results

### 3.1. Co-Design Workshops A: Initial Review and Feedback

#### 3.1.1. Procedure and Analysis

The next iteration of the CIRCLE framework was targeted at 5–12 and 12–18 year age ranges. A qualitative study including 125 teachers and other staff, focussing on best practices for use with older children with additional needs, was completed. Details of which are published elsewhere [30]. This study provided the detail for interventions, supports and strategies that would underpin development for older learners’ content.

Next, a series of ten co-design workshops applying interactive and participatory methods [31,32] were completed over a 2-year period. Seventy stakeholders participated. Participants in the workshops included the research team, specialist additional support for learning teachers, classroom teachers, specialist therapists, managers of education and health services, psychologists, medical doctors, and parents/carers. The research team included combined expertise in complex intervention development, education, rehabilitation, autism, occupational therapy, physiotherapy, and speech and language therapy.

A core group of stakeholders attended most of the workshops and acted as an Advisory Group with additional responsibilities for consultation, review and leadership. This core group comprised a senior local authority education manager, a senior additional support for learning manager, a senior educational psychologist, senior members of the additional support for learning service, a head teacher, class teachers and senior therapists from speech and language therapy and occupational therapy.

In the workshops, which included open discussion, small group work and brainstorming, participants were presented with updates and ideas for development of the CIRCLE resources. Facilitators led the workshops, and recorded notes. The aim of the workshops was to review the existing CIRCLE manual, gather feedback on current use, determine the validity of development ideas and develop new ideas. Detailed notes capturing each group’s discussion, how the participants tackled each activity, and feedback on the intervention ideas were produced and analysed. The analytic procedure drew on qualitative approaches of thematic and framework analysis involving familiarisation, identification of a thematic framework, coding of the data according to the framework, charting the themes, and mapping and interpreting the data [33,34,35,36]. To safeguard trustworthiness and transparency, peer checking was undertaken with colleagues about emergent ideas. The facilitators prepared detailed presentations to reflect group discussions. These presentations were given to the next workshop in the sequence. In this way, the analysis and outputs from each workshop were verified and built upon at the next workshop in sequence, leading to the design ideas (i.e., broad goals for the CIRCLE update) which became the new aspects of the framework and associated materials.

#### 3.1.2. Outputs

Overall utility of CIRCLE. Feedback from participants in the early workshops emphasised that the utility of CIRCLE was perceived to be high, indicating that the existing materials had a positive impact on staff and children in terms of improving the practices of teachers. Use of the resources were perceived to increase understanding of children’s needs and related supports. As a reference point for busy teachers, the current manuals were perceived as easy to use, clearly laid out, comprehensive and comprehensible. School leaders and experienced teachers reported that CIRCLE was a useful part of the strategy to increase the amount of support provided directly by teachers in schools, and to effectively deal with issues in schools via school pathways (prior to requiring external support). It was reported that using the resource had helped staff to shift towards meeting the needs of all children along the lines of a more inclusive classroom, rather than a “pull out” or “expert” model. CIRCLE resources were perceived to raise awareness of the inclusion “agenda.”

Uses in practice. As well as use by classroom teachers, specialist staff had been using the resource to share information between colleagues (e.g., at times of transition), or between the teacher and the additional support for learning services, or for meetings. The CIRCLE manual had been used as a resource by teachers to photocopy and tick strategies to try with individual pupils. The resources were also perceived to give teachers ideas as to what to do next, help to guide staff through the referral process for extra support, and in some cases prevent unnecessary referrals. It was also reported that there was a function for clear and transparent documentation about which strategies had been used in school.

Requirement for pencil and paper tools and other issues. Participants reported that they were frustrated that there was no formal method presented in the manuals to record information on children’s progress, and no formal way to communicate information using the existing CIRCLE framework. How to use the manuals to facilitate recording of information, and the associated tools required for this, was a key area of discussion. Participants reported that it would be helpful if the manual contained specific checklists and other pages, which could be photocopied and shared between teachers and related services personnel. Concerns around duplication of content across the resource were also reported. Descriptions and acronyms used in the resource were felt to be confusing, and some of the sections (particularly the inclusive classroom section) was perceived to be too short and lacking in content. Additionally navigation of the main document was described as problematic, and there were reported difficulties in finding the relevant sections to support a specific child.

Theoretical aspects. Presentation of a more coherent theoretical framework was requested. It was considered necessary to move the focus away from the child’s perceived deficits towards wider aspects of inclusion. It was felt that there should be more content related to inclusive environments. The overall presentation of strategies was to be reviewed, particularly to highlight the importance of environmental adaptations. The participants in the early workshops unanimously agreed the documents should be carefully written to reduce emphasis on child deficits. Throughout, it was felt terms should be replaced by positive headings wherever possible. Although the resources were largely felt to be clear, it was requested that a systematic process should be developed to facilitate use of the materials.

### 3.2. Co-Design Workshops B: Theoretical Framework

#### 3.2.1. Procedure and Analysis

The Medical Research Council guidance on development of complex interventions includes conceptual framework development as a necessary step [13]. The main ideas considered in the development of the CIRCLE framework focussed on person-environment interactions [37] and models of participation [38]. The primary theoretical framework used was the Model of Human Occupation (MOHO) [39]. The MOHO is a rehabilitation model that aims to enhance people’s participation, and includes concepts related to how people engage in everyday life focusing on values, attitudes, habits, routines, skills and the environment [39]. The relevance of the MOHO to understanding psychosocial and environmental factors in community, home and school situations for children is well-established [40,41,42,43,44]. The MOHO uses a holistic understanding of people, their daily life activities, interests and needs, and relationship with their environment to develop interventions [39]. People are conceptualised as being participation driven, with satisfactory engagement in personally and socially valued activities and roles seen as the fundamental outcome of interventions [39]. MOHO theory encourages interventions at multiple levels, and provides a structure for assessment and application of supports and strategies. It was agreed, due to its known utility for understanding the needs of children, and person-environment approach, to use the MOHO to guide the development of the CIRCLE framework. Using this model as the broad basis for CIRCLE meant several aspects had to be considered in tandem. The MOHO examines personal (i.e., motivation, habits and skills) and environmental (e.g., physical and social environment) influences [39]. This means understanding that “impairment” and “disability” are not person focussed, but linked to, and oftentimes driven by the environment. Adopting such a model is in response to critique around the labelling issues, stigma and disadvantage associated with a deficit or “medical” model approach [45]. A model that focuses on participation provides a beneficial alternative structure, meeting calls for a “social” or “biopsychosocial” approach [38,39].

Using MOHO theory as a guiding structure, common themes and features that related to the core intervention concepts were developed, discussed, modified, debated and eventually ratified in the co-design workshops. Across workshops, the focus was towards developing a framework that could be used to organise and present important ideas to the users, and how to “translate” MOHO concepts, some of which were unfamiliar to teachers, into a useable format and language. To our knowledge, this was the first application of these ideas for teachers. Using the above review, discussion and analysis led to the development of a new CIRCLE theoretical framework.

#### 3.2.2. Outputs

**Figure 2:**
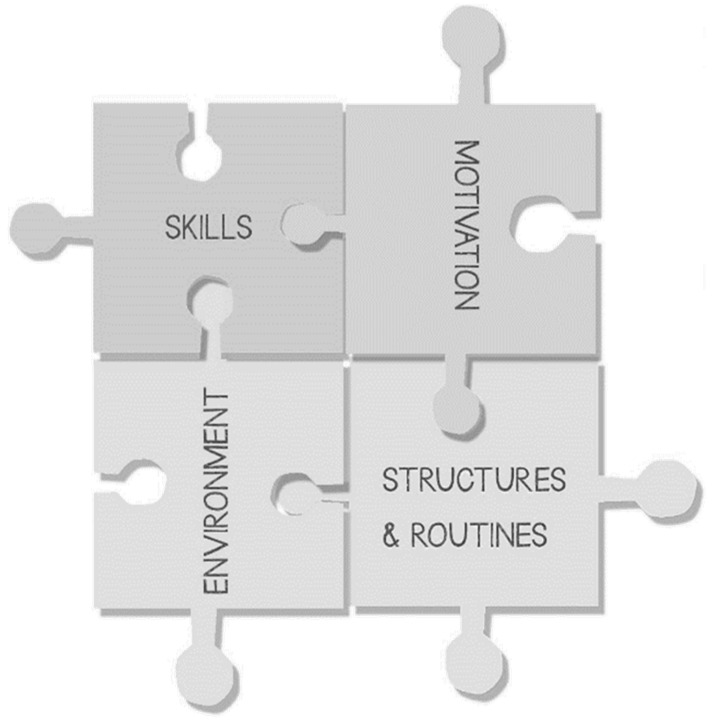
Updated CIRCLE framework. Reproduced from CIRCLE manuals [46,47].

The updated CIRCLE Framework, which encompasses a streamlined and education focussed application of MOHO concepts, presents children’s inclusion and participation in terms of four main areas (see Figure 2 and Table 1):

1. The environment (physical and social),
2. Structures and routines
3. Motivation
4. Skills

We presented the framework as a jigsaw puzzle to highlight the interconnectedness of the component parts. Workshop participants had perceived that there was a tendency by some teachers and others to focus on a child’s physical, sensory or behavioural deficits. They agreed that it was important to shift focus towards different areas (including environment and attitudes) rather than on deficits. Within this framework, it was important to stress some key ideas. Firstly, that participation and inclusion are a function of the child and environment together [38,39] with particular reference to the social model of disability [22]. We also took from MOHO the idea that people and environments are a complex dynamic system, and that children’s needs are best understood within a framework that considers motivation, structures and routines, and skills together. A key aspect identified was that the support for children should continue to take place in typical classrooms—and that this was the main and best place for most children. A key focus was therefore the environment. The idea of “environment first” was consistently highlighted as important by the workshop participants. Therefore, environmental adaptation and review of the environment should be the first step. Rather than thinking about what a learner can and cannot do, or only thinking about their underlying ability, the framework encourages teachers to think more widely, particularly paying attention to the disabling aspects of the environment. Using MOHO theory, the environment is understood to contain physical and social components, each of which requires attention [39].

Structures and routines were also included as an explicit aspect of the model [39]. Routines (i.e., daily structures of the school) support children to be able to anticipate transitions and required actions. Order and consistency are clearly helpful for children, and for some, school is the place where this is most apparent and helpful. For example, children may benefit from strategies including explicit structuring of the day/week or provision of visual supports (i.e., visual timetables) to help them follow routines or understand what comes next.

Motivation was included as an explicit aspect of the model, again drawing on MOHO theory. This focused across three overarching themes: “interests,” “values,” and “abilities.” Children’s “interests” is related to motivation and activities that are engrossing, enjoyable or satisfying to them. Supportive practices include utilising learners’ own specific ideas, hobbies, or cultural background, and ensuring individualisation and options. “Values” refers to what children find important and meaningful to them. Supportive practices include listening to and valuing children’s views, jointly setting goals, and self-assessment. Lastly, “abilities” refers to how children perceive themselves in terms of their ability and capacity. Supportive practices associated with this idea include differentiating work, setting achievable goals whilst ensuring challenge, giving positive feedback, and affirming interests, languages and cultures.

The presentation of supports and strategies was split into areas that were felt to be “neutral” labels (rather than condition specific or deficit focused). For younger learners these were: Attention and Concentration Skills; Organisational and Planning Skills; Posture and Mobility (Gross Motor) Skills; Dexterity and Manipulation (Fine Motor) Skills; Social, Emotional and Relationship Skills; and Verbal and Non-verbal Communication Skills. For older learners these were Attention and Concentration Skills; Organisation and Planning Skills; Motor Skills; Social, Emotional and Relationship Skills; Verbal and Non-Verbal Communication Skills. These categories and labels were agreed by the co-design workshop participants, who identified them as the main underlying areas of challenge experienced by children in schools. Each area was described and suggestions of strategies were developed using positive language, with culturally diverse examples. In line with the theoretical framework, throughout the manuals the strategies were split into “modifications to the learning environment” first, followed by “establishing structures and routines” second and “approaches to enhance motivation” third [for examples, see Supplementary Materials A5 and A6].

### 3.3. Co-Design Workshops C: Developing Pencil and Paper Tools

#### 3.3.1. Procedure and Analysis

To reflect the conceptual framework, and at the request of the stakeholders in the co-design workshops, pencil and paper tools to support information gathering and information sharing were developed. Two tools were developed: the CIRCLE Participation Scale (CPS) and the CIRCLE Inclusive Classroom Scale (CICS). These tools were designed to form the assessment portion of the CIRCLE intervention, to facilitate teachers’ engagement with the manual, and as a method for identifying where to target supports and strategies for children.

Workshop participants had indicated that teachers valued and tended to engage with tools that were quick and easy to use. They also reported that teachers were looking for tools that could be used to record input/change in order to support communication with colleagues, partner services and agencies, parents, and with the children themselves. Furthermore, a key output of the co-design workshops was to have tools that would shift teacher focus from children’s impairments towards one where they considered environmental factors first. It was also important to develop easy to use tools that would support teachers to consider their role in the inclusion of children, rather than considering this a role devolved to specialists.

The CPS and CICS were developed as first level tools to be used sequentially (environment tool first, followed by the participation tool). They were designed to be concise and easy to use by all education and related services personnel irrespective of training or level. To develop these tools, factors considered to have a potential influence on children’s participation were identified drawing on our previous research [12,30] and using the Model of Human Occupation (MOHO) [39] as a theoretical guide. To ensure content validity, face validity and utility, over the course of three meetings the co-design workshop Advisory Group reviewed and provided commentary. Items were identified for inclusion and exclusion at this point. In the next phase, a longstanding independent group (N = 9) of senior professionals was asked for further comment. This group included managers, educational psychologists and researchers. This group met four times. Based on these activities, pilot versions were designed. A pilot version of the CPS was field tested in three schools by teachers, who provided detailed feedback. Classroom teachers, head teachers and representatives from a parent organisation also provided feedback and commentary on the CICS. Comments helped the research team to clarify and revise the tools, including wording of items and instructions for use, before finalisation.

#### 3.3.2. Outputs

Circle Inclusive Classroom Scale (CICS): The CICS is a pencil and paper rating tool which identifies environmental barriers/supports to inclusion and participation. The tool comprises 3 domains developed using MOHO theory: the physical environment, the social environment, and structures and routines within the environment. Sub-domains for consideration include children’s participation in decision making, routines, appeal of activities, expectations, activity demands, empowerment, provision of information, relationships, support and facilitation, attitudes, availability of objects, visual supports, sensory space, and accessibility/adequacy of physical spaces. The CICS utilises a 4-point rating scale for all the items within each sub-domain. Using this scale, based on the judgement of the teacher completing the assessment, a “4” rating indicates a domain that strongly supports participation of learners, whereas a “1” rating indicates aspects that strongly interfere with participation of learners where improvement is required. As well as a rating sheet and recording format, the CICS also includes a set of reflective questions that help users when considering the quality of the classroom environment. Scoring is based on observation and a “walk through” of the classroom. The CICS can be completed by one individual, or by colleagues working together [see Supplementary Materials A7–A9 for examples of content].

CIRCLE Participation Scale (CPS): The aim of the CPS is to facilitate identification and measurement of factors impacting on children’s participation in school. The CPS was designed for use in general classrooms by teachers and other related staff for children aged 5–18 years with additional support needs, including physical, developmental or learning needs. The CPS consists of 10 short sections that cover the environment, structures and routines, motivation, and skills. The item pool contains items assessing the potential determinants of school participation across these areas. Each section of the CPS contains five items, which are positively framed statements with a 4 point Likert scale in which teachers rate the frequency with which, in their opinion, each item is observed for a given child. A lower score on the CPS indicates presence of barriers or needs for the child in that area, and potential requirement for additional support from staff. Once the CPS is completed, it directs users to relevant sections of the CIRCLE resources containing supports and strategies for teachers to try with children [see Supplementary Materials A7–A9 for examples of content].

Feedback: Feedback received on the tools indicated they were functional and straightforward to use. Teachers highlighted the benefit of tools that could be used for a broad range of children that directed them towards appropriate supports and strategies within the CIRCLE manuals. Feedback indicated they were a useful way to consider provision of supports for children and a useful way to share information between colleagues, e.g., between teachers at times of transition, or between the teacher and the specialist team, or at meetings. Specialist staff reported that the tools could be useful in supporting communication between classroom teachers and specialist teachers. It was felt that the CPS and CICS encouraged teachers to look at areas of the child (e.g., motivation) or the environment (e.g., peer support) that they might not have initially considered and encouraged teachers to complete assessment of the child and the environment. Although feedback was mainly positive, a few teachers expressed concerns. These included anxieties around the time that it might take to complete the tools and anxieties around the replacement of more comprehensive or specialised (e.g., dyslexia) assessments. Others were concerned that some of the items related to the environment were outside their control. Several suggestions were made on how the tools could be used to support collaborative working.

### 3.4. Design Specification and Building a New Manual

#### 3.4.1. Procedure and Analysis

This stage of the research developed the ideas and outputs from the previous manual and new research into new materials, principally new intervention manuals. Workshop participants were supportive of developing the manuals, as they stressed the usefulness of having useable, physical copies of the CIRCLE Framework (including the tools and supports/strategies) easily available in the classroom setting. To develop the new manuals a tendering process to identify a graphics design company was undertaken. The design brief and specification were included in the tender. Low-fidelity prototype documents were created in Microsoft publisher and sent to the contracted company. The research team worked with the contracted company using the following specification:

- Creative design of document elements (cover, tables, diagrams, lists, text styles, section navigation, glossary)
- To create a resource that is functional and easy to use
- To reduce reliance on text (e.g., design of diagrams) and improve use of colour
- To create a professional finish
- Improve readability

Following development of high-fidelity prototypes, a final co-design workshop took place in a university space, including several members of the target audience, i.e., teachers. Copies of the manuals were also sent to the Advisory Group and representatives from paediatric therapy services, head teachers, senior managers within the local authority, parents and experts in inclusive education, with feedback requested. Participants were provided with a draft copy of the manual and asked to “use” the manual with a child in mind with the aim of testing its usability and validity. Feedback, was recorded and transcribed. A list of required revisions was approved.

#### 3.4.2. Outputs

A list of revisions was developed. Issues were identified including document structure and signposting to relevant initiatives nationally and locally. Issues were attended to, and comments were fed back to the design company and a final version of the younger (5–12 years) [46] and older children’s (12–18 years) manual was published [47].

### 3.5. Naturalistic Implementation

#### 3.5.1. Procedure and Analysis

The newly developed manuals were distributed within the local authority who commissioned the work from 2016/2017 onwards. Across primary (elementary) and secondary (senior) provision this included c85 primary schools (5–12 years), c20 secondary schools (12–18 years), and c3000 teachers.

The roll out of the resources was managed by a senior member of the Additional Support for Learning Service. To facilitate training, a senior educational psychologist also worked with colleagues to develop a training structure for teaching staff, focused on self-evaluation and using the various tools in the resources effectively. The training and implementation was fully led by the local authority. Designing the implementation in this way ensured that it was absorbed into naturally occurring practices. This aspect was designed to foster informal networks, to leverage existing organisational structures, to ensure that knowledge of the framework and ability to train and educate using the framework was distributed, and to have the change be led by insiders rather than external “experts.” This ensured that the implementation was resilient and robust, and met the needs of the target community.

Once the manuals had been made available to schools, and training completed, we collected feedback from individuals representing a convenience sample of 10 schools (staff from 8 primary schools and 2 secondary schools), as well as from a group of 20 senior professionals including senior managers, head teachers, and senior additional support for learning teachers.

#### 3.5.2. Outputs

Evaluation of implementation reach across the authority. After approximately 18 months, all schools had been trained and were in possession of CIRCLE manuals. Specialist additional support for learning staff had also received training and were given resources with agreement that they would train additional staff as required. One copy each of the relevant resource had been printed for all teachers in the local authority (primary, secondary and special). Additional arrangements for further training were that leadership and support for learning teachers could attended specialist training, and cascade this training as appropriate. All teaching staff were expected to have an awareness of the resource and use it in their practice. Use in formal local authority processes was instigated, including requests from schools for extra support for children, school self-evaluation and school policies for inclusion. CIRCLE implementation was therefore supported by senior management within the authority and through strategic planning, making CIRCLE a core tool for the authority that teachers were expected to engage with. Several schools reported using CIRCLE as part of formal processes, including writing individual education plans, referrals to specialist providers, and communication with parents. Some schools reported making the use of the CICS a feature of their school calendar, where teachers carried the assessment out at the start of a new school year, ensuring that all started the year engaged with the CIRCLE framework. However, implementation between schools was varied. Schools reported that implementation was more robust where training had been completed as a ‘whole school’ strategy, rather than led by individuals. Some schools reported that implementation was still in the early stages.

Feedback from users. The CIRCLE resources have been well received in terms of usefulness, structure and ease of use. CIRCLE was reported to be an effective first level universal intervention for new staff, as well as a guide for more experienced staff. Teachers reported that use of the manuals supported them to clearly articulate strategies that they may include as part of their routine practice. This encouraged teachers to focus on their own individual responsibility for pupils within their class and provided a framework for discussions with other teachers and colleagues. The resources were felt to support teachers and related services personnel to look at areas of the child and environment that they might not have initially considered—and encourage teachers to do “detective work” around the child and what could help that child. The parts of the resource most commonly used were the CIRCLE Inclusive Classroom Scale, the CIRCLE Participation Scale and the various Supports and Strategies pages. Most feedback about these sections was positive, but some teachers reported that the resource, whilst helpful, was long, and required time to read and understand.

A few teachers also reported that they found it challenging to complete the tools without discussion with others. Some secondary school teachers highlighted that they did not have their own specific teaching space, so that they perceived that they had little control over many aspects of the environment. Concern was also expressed that some teachers were reluctant to change some aspects of the environment to suit one or a small group of children if they thought that there was no benefit for the larger group. Additionally it was noted that the process, as recommended in the manual, would be difficult to complete at the start of a new session/school year, as the class would be new at that point, so that the teacher would not know the children. Not unexpectedly, a few teachers reported concerns around the time taken to complete the tools possibly in addition to other assessments; however, others thought they could be completed in a timely manner.

## 4. Discussion

The development process described in this paper included qualitative research, stakeholder involvement and naturalistic implementation, to develop, refine and implement a complex intervention.

Principles underlying the CIRCLE intervention are that activities and interventions should be collaborative, preventative, high frequency, and based in the classroom. The thinking on this approach is that interventions should be embedded in daily lessons and routines, provided by classroom teachers as part of routine practice, rather than creating an isolated strategy or event, provided by “experts.” High frequency refers to use in the daily routines of the class. Given the opportunity to provide this first level support in the natural environment of the classroom, this might reduce the need for specialist services (i.e., the CIRCLE intervention might be preventative). The CIRCLE intervention supports collaborative working with others as required, by providing a common framework and language to support communication and discussion with a range of education and health staff, parents and the child.

Within CIRCLE, the child’s difficulties are not the primary concern and a combination of strategies, in particular those focusing on the environment, are required. Rather than thinking about what a learner can and cannot do, the CIRCLE Framework encourages teachers to consider what affects learner’s inclusion and participation within the context of the environment, and to take responsibility for “delivering” inclusion within their classroom. Such approaches support professionals to embrace the complexity of the child’s needs, create a “working hypothesis” about a child’s situation and provide transparency of reasoning. They also support a consistency of service provision and provide a common language for communication and research.

It is likely that a key mechanism underpinning CIRCLE is focused discussions on inclusion. It is possible that other frameworks with similar aims, but developed with different content in different ways, might also achieve similar outcomes. Although this may be the case, it remains true that there is considerable debate in the field on the best methods of achieving inclusion-focused change [23,24,25,26,27]. A framework underpinning inclusion must be clear and unambiguous in order to support understanding, improve practices and influence policies. Within the CIRCLE framework, a key strength is the clarity and combination of components across child and environment.

With reference to other approaches, there are several other inclusion-focused frameworks [48,49,50,51]. However, these have tended to focus on measurement of whole school inclusive practice [49], theoretical principles [50], or require a whole system approach [51]. It is arguably the case that previous efforts to develop inclusion-focused frameworks have sometimes involved “ivory-tower” efforts failing to take account of stakeholder perspectives and real-world problems faced by schools. Our research suggests that what teachers require is a practical framework that is easy to understand, with supports and strategies for use in the classroom, which are applicable to the range of needs and abilities that are increasingly common in schools. Reflection on practice, a common language and a structure for communication and information sharing are also important. As noted, there is a very significant literature on ideas around inclusion. Along with the extensive range of different specialised interventions, this makes application for teachers, who have a general responsibility for an age-based cohort with varying support needs, difficult. The CIRCLE framework is an intervention that any teacher can use. The framework has been designed with significant collaboration from stakeholders, including teachers and school leadership, with excellent ecological and face validity, and proven feasibility for use in the school context. This meets the need for a universal inclusion focused framework that can be applied in schools as a first level support.

Within the CIRCLE framework, the CICS and CPS pencil and paper measurement tools were developed to provide individual teachers, departments or schools a means of assessing the environment and children’s participation, and of documenting and sharing this. The CICS was intended to allow the user to formally rate the classroom. Together with strategies oriented for developing an inclusive classroom, this was designed to encourage focus on the environmental aspects of inclusion. The CPS was considered the next step and intended to enable identification of a learner’s strengths and also areas requiring development. It was designed to help identify which groups of supports and strategies to try by directing the user to further specific sections within the CIRCLE manual. It is a truism noted within any system, including education, that what is measured will become what is done. Therefore, the CPS and CICS provide an avenue for encouraging a beneficial focus on inclusion across child and environment.

A final issue of interest concerns the role of external specialists, who may be part of the multidisciplinary team involved with schoolchildren. Many countries have multidisciplinary and multiagency teams involved with diagnosis, assessment and intervention. A key challenge is engagement of these teams across services; particularly as inclusion and participation in school is increasingly accepted as an important component of a holistic health intervention [52]. Recent priority setting exercises for children with learning difficulties [53] and neurodisability [54] have emphasised the importance of schools and teachers in terms of training, identifying optimal learning environments and facilitating interagency collaboration. There is therefore inherent value in working with and considering the views of multiple professional groups. External professionals collaborating with teachers can support ecological assessment and intervention, as well as facilitating a focus on school participation within their own practice. CIRCLE could provide a framework and language to support discussions between external staff and education staff around implementation of first level supports for children.

### 4.1. Recommendations

Since the initial evaluation, the CIRCLE manuals continue to be used within schools in the local authority where the research was originally carried out. The CIRCLE manuals have also been adopted in other areas across Scotland. Feedback indicates positive influence in terms of practice, through improved teacher confidence and competence in delivering early interventions and first level supports. To support dissemination, all CIRCLE manuals are freely available online [55]. To accompany the materials, a detailed list of recommendations for implementation across different school levels and individual roles was developed (Table 2). A key emphasis of CIRCLE is that teachers take responsibility for inclusion, without overt focus on specific diagnoses or needs. However, it is known that children with a range of needs for example autistic children, have particular needs which require greater understanding and more focused interventions. Use of CIRCLE represents the first universal level of intervention that should be in place. This will support all children, as well as providing a basis for support of children with more specific needs. Such first level support comprises core information and key messages required by all staff. With this foundation in place, training in more specific evidence based approaches or ways of working is still recommended. Using CIRCLE will provide staff with a foundation on which to build their knowledge and skills around supporting children with additional needs in schools.

### 4.2. Limitations

This paper focusses on the processes used to develop a complex intervention. The preliminary findings are positive as outlined, providing good data on adequacy of theoretical underpinnings and feasibility and acceptability of CIRCLE in a variety of real-world conditions. However, although there has been extensive stakeholder involvement, feedback represents a convenience sample, which may not have been representative of the views of all teachers in the authority. Additionally, it is beyond the scope of this paper to discuss investigation into the efficacy of the CIRCLE intervention in terms of outcomes for children and young people. Future study will be required to ascertain these wider outcomes.

## 5. Conclusions

Building application of inclusion-fostering theory and interventions into practice is fundamentally important. In this research, the Medical Research Council guidance on the development of complex interventions has been used to improve and implement a new intervention. The methods and processes undertaken mean that professionals, academics and experts with a wide range of backgrounds and expertise have been involved, including teachers, educational psychologists, a range of specialist teachers, and senior representatives from children’s therapy services. The research provides a useful and novel example of developing a complex intervention in schools including co-design and stakeholder involvement.

## Figures and Tables

**Table 1:** Overview of CIRCLE framework components *.

Framework Component	Description
Physical environment	The physical environment refers to the physical layout of the classroom and the resources used within it.
Social environment	The social environment is concerned with the attitudes, expectations and actions of those within the class and how these can affect children either positively or negatively.
Structures and routines	Structures and routines are events that happen in the same way with regularity. The start, middle and end of the routine becomes predictable through repetition.
Motivation	Motivation gives children incentive, enthusiasm and interest when engaging with activities and the people around them. Children are motivated by their own feelings, desires, self-esteem and confidence.
Skills	Skills refer to a learner’s skills in the key areas: attention and concentration; organisation and planning; posture and mobility; dexterity and manipulation; socialising, emotions and relationships; verbal and non-verbal communication.

* reproduced from CIRCLE manuals [46,47].

**Table 2:** Recommendations for implementation of CIRCLE across various levels of practice.

Group	Recommendations for Implementation
Classroom Teachers	Download the CIRCLE manual Complete CIRCLE Inclusive Classroom Scale each term Use CIRCLE assessment processes and CIRCLE supports and strategies with specific children Use CIRCLE paperwork for communication and collaboration with colleagues in school Know how and when to ask for support Use photocopied CIRCLE pages ticked around what supports and strategies have already put in place at point of referral/request for support Use CIRCLE assessments in collaborative working with parents and other partners
Specialist teachers, senior teachers, psychologists, health and therapy staff	Download the CIRCLE manual Encourage teachers to download CIRCLE manual Regularly encourage teachers to complete CIRCLE processes before seeking out support Routinely ask for CIRCLE paperwork (e.g., printed sheet with ticked off supports, classroom assessment or child assessment) when working with teachers Promote the ‘Inclusive Classroom’ as a core strategy in school Regularly encourage teachers to take increased responsibility for inclusive practice and anticipatory supports Use CIRCLE resources in communication and collaborative working with parents and partner services Use CIRCLE in providing training to teachers on universal supports
School Leadership	Understand and promote CIRCLE across the school Use CIRCLE as part of student teacher and new teacher induction Encourage staff to download the CIRCLE manual Provide funding to print manuals and materials for ease of use Reference CIRCLE in school policy and school web-site Use CIRCLE to provide professional learning for staff on universal supports Promote CIRCLE as first step pf professional learning pathway for developing knowledge and skills Use CIRCLE paperwork in school processes Promote the ‘Inclusive Classroom’ as a core strategy in school Use the CIRCLE Inclusive Classroom Scale in quality assurance and/or audit processes Use CIRCLE paperwork to support referrals to partner services and other agencies Use CIRCLE resources to support communication and collaborative working between school staff, parents and partner services
Area or local government	Published Policies and plans reference CIRCLE and encourage the use of CIRCLE materials CIRCLE paperwork is used in relevant official processes (e.g., referrals for extra support) Government or “official” web-site references CIRCLE All relevant staff are aware of CIRCLE resources Recommend CIRCLE as the first step of professional learning pathway for developing knowledge and skills Ensure that professional learning refers to CIRCLE

## Data Availability

Please contact the corresponding author for study data. Data are not publically available due to confidentiality and ethical requirements. The Scottish Government Pupil Census Supplementary Statistics data are available online: https://www.gov.scot/publications/pupil-census-supplementary-statistics.

## Supplementary Materials

The following are available online at https://www.mdpi.com/2227-9067/8/3/217/s1, Supplementary Material A1 Pupils in primary mainstream schools, 2007–2019 Scotland. Supplementary Material A2 Pupils in secondary mainstream schools, 2007–2019, Scotland. Supplementary Material A3 Pupils in primary mainstream schools, 2012, 2017, 2019, Local Authority where CIRCLE was implemented. Supplementary Material A4 Pupils in secondary mainstream schools, 2012, 2017, 2019, Local Authority where CIRCLE was implemented. Supplementary Material A5 CIRCLE manual examples ages 12–18 year (4x pages). Supplementary Material A6 CIRCLE manual examples ages 5–12 years (4x pages). Supplementary Material A7 Domains, sub-domains and examples of from CIRCLE Inclusive Classroom Scale (CICS). Supplementary Material A8 Circle Inclusive Classroom Scale (CICS) reflective question examples. Supplementary Material A9 Overview of the CIRCLE Participation Scale (CPS).
