# Supplementary material for: Using Stakeholder Involvement, Expert Knowledge and Naturalistic Implementation to Co-Design a Complex Intervention to Support Children’s Inclusion and Participation in Schools: The CIRCLE Framework
Source: Children (Basel). 2021 Mar 11;8(3):217. doi: 10.3390/children8030217 (PMC8001448; doi:10.3390/children8030217)
Supplement: Supplementary file 1 [file children-08-00217-s001.pdf]

Supplementary Materials

Supplementary Material A1 Pupils in primary mainstream schools, 2007-2019 Scotland 2
Supplementary Material A2 Pupils in secondary mainstream schools, 2007-2019, Scotland 3
Supplementary Material A3 Pupils in primary mainstream schools, 2012, 2017, 2019, Local Authority where CIRCLE was implemented 4
Supplementary Material A4 Pupils in secondary mainstream schools, 2012, 2017, 2019, Local Authority where CIRCLE was implemented 5
Supplementary Material A5 CIRCLE examples ages 12-18 year (4x pages) 6
Supplementary Material A6 CIRCLE examples ages 5-12 years (4x pages) 11
Supplementary Material A7 Domains, sub-domains and examples of from CIRCLE Inclusive Classroom Scale (CICS) 16
Supplementary Material A8 Circle Inclusive Classroom Scale (CICS) reflective question examples 17
Supplementary Material A9 Overview of the CIRCLE Participation Scale (CPS) 18

Supplementary Material A1 Pupils in primary mainstream schools, 2007-2019 Scotland

	2007	2008	2009	2010	2011	2012	2013	2014	2015	2016	2017	2018	2019
**Total school roll**	375,946	370,839	367,146	365,326	366,429	370,680	377,382	385,212	391,148	396,697	400,312	400,276	398,794
**Total pupils with any ASN [1]**	16,478	17,626	19,881	34,165	50,985	62,572	69,513	74,327	79,839	88,517	94,125	101,558	107,635
**Percentage of school roll**	4.4	4.8	5.4	9.4	13.9	16.9	18.4	19.3	20.4	22.3	23.5	25.4	27.0
**Estimated pupils with Neurodevelopmental ASN [2]**	9,580	10,247	11,558	19,862	29,641	36,377	40,412	43,211	46,415	51,461	54,721	59,042	62,575
**Percentage of school roll**	2.6	2.8	3.2	5.4	8.1	9.8	10.7	11.2	11.9	13.0	13.7	14.8	15.7

[1] **Scottish Government Schools census [1] - Any ASN defined as:** Learning disability; Dyslexia; Other specific learning difficulty; Other moderate learning difficulty; Visual impairment; Hearing impairment; Deaf blind; Physical or motor impairment; Language or speech disorder; Autistic spectrum disorder; Social, emotional and behavioral difficulty; Physical health problem; Mental health problem; Communication Support Needs; Interrupted learning; English as an additional language; Looked after; More able pupil; Young carer; Bereavement; Substance misuse; Family issues; Risk of exclusion

[2] **Estimated - Neurodevelopmental ASNs are defined as:** Learning disability; Other specific learning difficulty (e.g. numeric); Other moderate learning difficulty; Language or speech disorder; Autistic spectrum disorder; Social, emotional and behavioural difficulty; Mental health problem; Communication support needs.

Estimation methodology:
Neurodevelopmental ASNs are estimated. These categories are not presented by the Scottish government in their publically available data. In order to estimate Neurodevelopmental ASNs we used pupil level data collected from a random sample of primary schools in one large urban Local authority in Scotland (n=668 children) [2] where the proportion of Neurodevelopmental cases was identified. Using this proportion we are able to estimate Neurodevelopmental cases across Scotland in the period 2007-2019.

[1] Scottish Government. Pupil census: supplementary statistics 2007-2019. Available from https://www.gov.scot/publications/pupil-census-supplementary-statistics/ [accessed 15/10/2020]

[2] Report details available from the authors on request

Supplementary Material A2 Pupils in secondary mainstream schools, 2007-2019, Scotland

	2007	2008	2009	2010	2011	2012	2013	2014	2015	2016	2017	2018	2019
**Total school roll**	309,560	303,978	302,921	301,007	297,109	293,562	289,164	284,762	281,939	280,983	281,993	286,152	292,063
**Total pupils with any ASN [1]**	13,355	14,334	17,623	28,622	40,565	48,486	55,124	59,234	66,433	75,257	82,712	90,685	101,130
**Percentage of school roll**	4.3	4.7	5.8	9.5	13.7	16.5	19.1	20.8	23.6	26.8	29.3	31.7	34.6
**Estimated pupils with Neurodevelopmental ASN [2]**	7,764	8,333	10,245	16,640	23,583	28,188	32,047	34,436	38,622	43,752	48,086	52,721	58,793
**Percentage of school roll**	2.5	2.7	3.4	5.5	7.9	9.6	11.1	12.1	13.7	15.6	17.1	18.4	20.1

[1] **Scottish Government Schools census [1] - Any ASN defined as:** Learning disability; Dyslexia; Other specific learning difficulty; Other moderate learning difficulty; Visual impairment; Hearing impairment; Deaf blind; Physical or motor impairment; Language or speech disorder; Autistic spectrum disorder; Social, emotional and behavioral difficulty; Physical health problem; Mental health problem; Communication Support Needs; Interrupted learning; English as an additional language; Looked after; More able pupil; Young carer; Bereavement; Substance misuse; Family issues; Risk of exclusion

[2] **Estimated - Neurodevelopmental ASNs are defined as:** Learning disability; Other specific learning difficulty (e.g. numeric); Other moderate learning difficulty; Language or speech disorder; Autistic spectrum disorder; Social, emotional and behavioural difficulty; Mental health problem; Communication support needs.

Estimation methodology:
Neurodevelopmental ASNs are estimated. These categories are not presented by the Scottish government in their publically available data. In order to estimate Neurodevelopmental ASNs we used pupil level data collected from a random sample of primary schools in one large urban Local authority in Scotland (n=668 children) [2] where the proportion of Neurodevelopmental cases was identified. Using this proportion we are able to estimate Neurodevelopmental cases across Scotland in the period 2007-2019.

[1] Scottish Government. Pupil census: supplementary statistics 2007-2019. Available from https://www.gov.scot/publications/pupil-census-supplementary-statistics/ [accessed 15/10/2020]

[2] Report details available from the authors on request

Supplementary Material A3 Pupils in primary mainstream schools, 2012, 2017, 2019, Local Authority where CIRCLE was implemented

	2012	2017	2019
**Total school roll**	25,887	30,507	30,878
**Total pupils with any ASN [1]**	4,458	6,763	11,278
**Percentage of school roll**	17.2	22.2	36.5
**Estimated pupils with Neurodevelopmental ASN [2]**	1,718	2,606	4,345
**Percentage of school roll**	6.6	8.5	14.1

[1] **Scottish Government Schools census [1] - Any ASN defined as:** Learning disability; Dyslexia; Other specific learning difficulty; Other moderate learning difficulty; Visual impairment; Hearing impairment; Deaf blind; Physical or motor impairment; Language or speech disorder; Autistic spectrum disorder; Social, emotional and behavioral difficulty; Physical health problem; Mental health problem; Communication Support Needs; Interrupted learning; English as an additional language; Looked after; More able pupil; Young carer; Bereavement; Substance misuse; Family issues; Risk of exclusion

[2] **Estimated - Neurodevelopmental ASNs are defined as:** Learning disability; Other specific learning difficulty (e.g. numeric); Other moderate learning difficulty; Language or speech disorder; Autistic spectrum disorder; Social, emotional and behavioural difficulty; Mental health problem; Communication support needs.

Estimation methodology:
Neurodevelopmental ASNs are estimated. These categories are not presented by the Scottish government in their publically available data. In order to estimate Neurodevelopmental ASNs we used pupil level data collected from a random sample of primary schools in one large urban Local authority in Scotland (n=668 children) [2] where the proportion of Neurodevelopmental cases was identified. Using this proportion we are able to estimate Neurodevelopmental cases across Scotland in the period 2007-2019.

[1] Scottish Government. Pupil census: supplementary statistics 2007-2019. Available from https://www.gov.scot/publications/pupil-census-supplementary-statistics/ [accessed 15/10/2020]

[2] Report details available from the authors on request

Supplementary Material A4 Pupils in secondary mainstream schools, 2012, 2017, 2019, Local Authority where CIRCLE was implemented

	2012	2017	2019
**Total school roll**	18,505	18,503	19,772
**Total pupils with any ASN [1]**	3,391	5,421	8,117
**Percentage of school roll**	18.3	29.3	41.1
**Estimated pupils with Neurodevelopmental ASN [2]**	1,307	2,089	3,127
**Percentage of school roll**	7.1	11.3	15.8

[1] **Scottish Government Schools census [1] - Any ASN defined as:** Learning disability; Dyslexia; Other specific learning difficulty; Other moderate learning difficulty; Visual impairment; Hearing impairment; Deaf blind; Physical or motor impairment; Language or speech disorder; Autistic spectrum disorder; Social, emotional and behavioral difficulty; Physical health problem; Mental health problem; Communication Support Needs; Interrupted learning; English as an additional language; Looked after; More able pupil; Young carer; Bereavement; Substance misuse; Family issues; Risk of exclusion

[2] **Estimated - Neurodevelopmental ASNs are defined as:** Learning disability; Other specific learning difficulty (e.g. numeric); Other moderate learning difficulty; Language or speech disorder; Autistic spectrum disorder; Social, emotional and behavioural difficulty; Mental health problem; Communication support needs.

Estimation methodology:
Neurodevelopmental ASNs are estimated. These categories are not presented by the Scottish government in their publically available data. In order to estimate Neurodevelopmental ASNs we used pupil level data collected from a random sample of primary schools in one large urban Local authority in Scotland (n=668 children) [2] where the proportion of Neurodevelopmental cases was identified. Using this proportion we are able to estimate Neurodevelopmental cases across Scotland in the period 2007-2019.

[1] Scottish Government. Pupil census: supplementary statistics 2007-2019. Available from https://www.gov.scot/publications/pupil-census-supplementary-statistics/ [accessed 15/10/2020]

[2] Report details available from the authors on request

Supplementary Material A5 CIRCLE examples ages 12-18 year (4x pages)

CIRCLE documents are available at

https://www.thirdspace.scot/circle/education-resources

Verbal and Non-verbal Communication Skills

Learner may require support with expressive or receptive aspects of communication.

Learner may require support with:

- ☐ Understanding, retaining and carrying out instructions
- ☐ Understanding and using conceptual language
- ☐ Understanding, using and recalling vocabulary and/or complex grammar that is heard or read
- ☐ Clearly expressing thoughts/ideas, verbally or in written form
- ☐ Pronouncing sounds and words and/or speaking fluently
- ☐ Social interaction, including social use of language (pragmatics) - for example, being aware of non-verbal cues such as body language, facial expression and what is being inferred
- ☐ Behaviour that may be related to frustration because of communication difficulties
- ☐ Listening

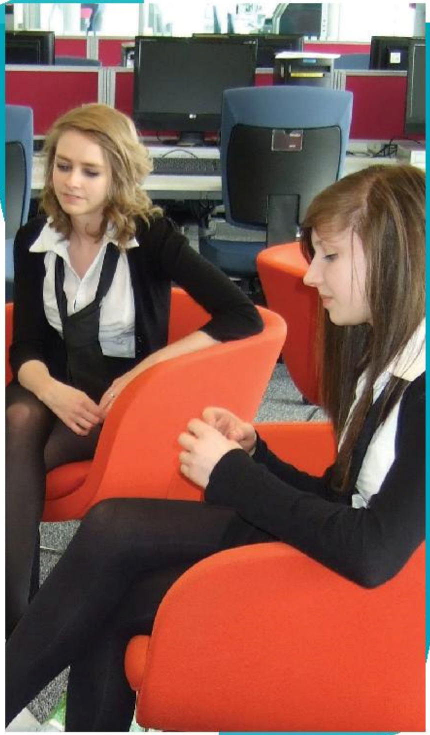

Verbal and Non-verbal Communication Skills

 Copyright © 2020

CHILD INCLUSION RESEARCH INTO CURRICULUM LEARNING EDUCATION

Verbal and Non-verbal Communication Skills

Principle:

Adapting teacher's own communication style; provision of structures and environmental modifications that facilitate learners' communication, literacy and social communication skills.

- ☐ Adapt and use your own communication to facilitate that of others.
- ☐ Provide opportunities and resources that develop and support communication and social interaction skills.
- ☐ Think about how the environment (physical, sensory and social) can be used to facilitate effective communication and to support understanding.

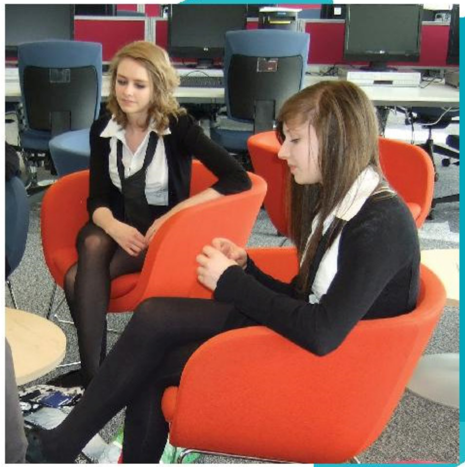

"Every now and again I have to step back and think about the vocabulary that I'm using. I know that at times I've lost learners because they don't understand, and that comes across when they ask something basic - maybe some sort of word that you'd have expected them to know, but they don't."

"Break times and other unstructured times are very difficult because of all the social communication parameters that are involved. For some, break times can be the most challenging aspect of the whole day."

See www.thirdspace.scot/circle for more resources including interventions and practical supports.
Copyright © 2020

Verbal and Non-verbal Communication Skills

Strategies

Modification to the Learning Environment

- ☐ Be aware of vocabulary demands of tasks. Teach and reinforce new or unfamiliar vocabulary as it arises.
- ☐ Consider reducing the rate and pace of lesson delivery and reducing language demands of tasks - for example, by decreasing length of sentences used in oral instruction and/or written texts.
- ☐ Ensure your language is clear and that it is consistent when explaining concepts.
- ☐ Explain complex language or concepts and illustrate with examples.
- ☐ Supplement verbal instructions with visual cues and demonstration.
- ☐ Use peer mentoring and buddy systems within class.
- ☐ Consider seating arrangements (proximity to supportive peers, proximity to teacher, position in relation to windows and doors, and consistency of seating).
- ☐ Use visual prompts and cues within the classroom setting to promote understanding, such as visual timetables and pictorial representation of subjects.
- ☐ Where possible, provide a calm classroom environment, keeping disruptions to a minimum.
- ☐ Use of reader/scribe/ICT as appropriate.
- ☐ Take into account sensory preferences and be aware of how sensory factors such as noise or visual stimuli may affect communication and interaction.

Establishing Structures and Routines

- ☐ Use visual timetable to support understanding of predictable routines and reduce anxiety.
- ☐ Use visual cues to support understanding of changes to routines to reduce anxiety.
- ☐ Provide 'get out of class early' passes for those who find transition times, such as between classes and end of day, difficult*.
- ☐ Use consistent, clear classroom procedures, and prepare fully for any changes to regular routines.
- ☐ Check understanding regularly by asking learners to summarise what they have heard.
- ☐ Encourage use of word banks, subject glossaries and personal dictionaries.
- ☐ Use ICT that supports literacy (e.g. specific computer programmes for literacy).
- ☐ Provide support at break times to support social communication*.
- ☐ Ensure homework is written down (consider giving printed copy).
- ☐ Set clear expectations of what is required in a writing task (e.g. full sentences or bullets, specific headings, length of piece, etc.).
- ☐ Use consistent roles in group work (e.g. note maker, reporter, discussion leader, etc.).

Approaches to Enhance Motivation

- ☐ Consider literacy demands and differentiate appropriately to ensure engagement.
- ☐ Facilitate use of first language when appropriate.
- ☐ Use learners interests when designing tasks.
- ☐ Encourage learners to monitor their own understanding and to ask for clarification when needed.
- ☐ Use effective questioning - for example, consider using graded and targeted questions. Give adequate 'thinking time' before expecting a verbal response.
- ☐ Use experiential or active learning techniques, such as building models or drawing, to encourage engagement.
- ☐ Use reinforcement, repetition and rephrasing.
- ☐ Facilitate positive social interaction (such as peer mentoring) or by building on learners own social groups within the wider school*.
- ☐ Give general encouragement for desired communication.
- ☐ Practise and prepare for change and/or new experiences.

See www.thirdspace.scot/circle for more resources including interventions and practical supports. Copyright © 2020

*See page 70

Supports and Strategies Planning Page

CHILD INCLUSION RESEARCH INTO CURRICULUM LEARNING EDUCATION

Learners Name: ____________________ Completed by: ____________________

Class: ____________________ Position: ____________________

Date: ____________________

Summary of concerns from CPS:	Learner's views:
•	•
•	•
•	•

Most successful strategies used:	Learner's views:
•	•
•	•
•	•

Strategies to implement or use more consistently:	Learner's views:
•	•
•	•
•	•

Plan (What, How, Who, When)	Learner's views:
•	•
•	•
•	•

Outcome:	Learner's views:
•	•

Next Steps:	Learner's views:
•	•

See www.thirdspace.scot/circle for more resources including interventions and practical supports. Copyright © 2020

Supplementary Material A6 CIRCLE examples ages 5-12 years (4x pages)

CIRCLE documents are available at

https://www.thirdspace.scot/circle/education-resources

DEXTERITY & MANIPULATION (FINE MOTOR) SKILLS

"I first noticed that **Katie** had difficulty with her fine motor skills…things like doing buttons up, writing, cutting and colouring in. It was a big effort for her. When she was writing it was like her brain was working faster than her hand. So although she had good ideas she could not get them down on paper."

Learners may need support with coordinating their hands to perform functional tasks such as dressing (e.g. fastening buttons), using learning materials and tools (e.g. pencils), or manipulating objects (e.g. toys).

"**Lucja** enjoys most activities but does not get on with pen and paper. When talking she is fluent and her understanding seems good…but when she has to write something her ability level is not reflected and her confidence drops. She has a strange pencil grip which we are working on. She seems to do much better when we do active learning or when I use the interactive whiteboard. Using different media keeps her focussed and makes the task more enjoyable and accessible for her."

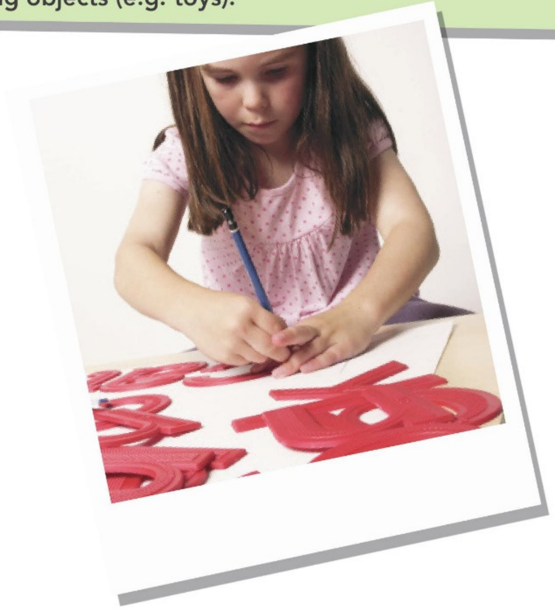

See www.thirdspace.scot/circle for more resources including interventions and practical supports. Copyright © 2020

DEXTERITY & MANIPULATION (FINE MOTOR) SKILLS

Principle

Develop dexterity and manipulation using clear instruction, task adaptation and environmental supports.

- Consider the learning environment and resources required to support dexterity and manipulation
- Provide clear instructions of the task and regular opportunity for practice
- Ensure tasks are adapted to provide opportunity to experience success

Parents say...

He's messy and clumsy.

He can't fasten his shoe laces or zip his jacket

She can't hold a pencil.

She can't use a knife and fork together."

Do you think he has dyspraxia?

Education staff suggest...

"For **Katie** … we use things like stencils for letters, different sizes and types of pencils and crayons and I've made specific worksheets for practice of letter formation - I make sure they are things that she can complete and use a sticker chart as an incentive. She also attends a small skills group 3 times a week where they work on hand strength - things like manipulating putty, squeezing close pegs, scrunching paper etc. - she really enjoys it. "

"As **Lucja** is in P7 we are now focussing on developing keyboard skills so that she can use ICT to keep up with peers in secondary school - she still struggles a bit but finds it an easier way to put ideas down on paper. I also give printed hand-outs or worksheets to minimise lengthy note taking."

"**Edward** struggles with using his hands for things like doing buttons and writing. We have tried using different pencil grips and found one which seemed to help, but he still tended to avoid writing tasks. I have recently set up a small group project where I have given him the role of project manager. He has really good ideas which he enjoys sharing with his peers and having them scribed. I have also provided a template to record bullet points from the project and he is now making more attempts to do some of the written work himself. He seems much happier and his self-esteem is being improved by the positive reinforcement he is receiving from his peers."

See www.thirdspace.scot/circle for more resources including interventions and practical supports. Copyright © 2020

DEXTERITY & MANIPULATION (FINE MOTOR) SKILLS
SUGGESTED SUPPORTS AND STRATEGIES

Modifications to the Learning Environment

- Seating arrangements and positioning in class e.g. to ensure learner sits correctly positioned and orientated with chair pulled well into desk
- Suitable furniture e.g. correct height of chair/desk or angled writing board
- Physical supports, equipment, aids and adaptations e.g. a range of different widths, weights and textures of pens/pencils/crayons, or a chunky stylus for use with a tablet.
- Hand over hand support e.g. guide the learner's hands while they fasten their jacket
- Tactile cues and supports e.g. tracing letters in sand
- Demonstration e.g. how to catch a ball
- Break tasks down
- Additional verbal instructions to support task e.g. talk through tying their lace as they do it
- Visual cues and supports e.g. use stencils to aid letter formation
- Modify tasks specific to learner's ability
- Offer compensatory methods e.g. use of laptop or scribing
- Collaborate with parents/carers

Establishing Structures and Routines

- Verbal rehearsal e.g. talking through how to tie shoe laces prior to doing so
- Use kinaesthetic learning routinely
- Practise desired skills with regular monitoring
- Regular specialist groups to develop dexterity and manipulation skills
- Templates e.g. to reduce the volume of written work required
- Allow extra time for completion of tasks
- Reduce the amount of work required
- Handwriting programmes
- Movement breaks e.g. 'warm up' of hands by pushing them together prior to activity
- Use routine peer support for specific activities

Approaches to Enhance Motivation

- Adapt tasks to ensure success e.g. allow learner to sit down when putting on their coat
- Use backward chaining to allow learner to experience success
- Use multisensory approaches to engage interest e.g. modelling with putty
- Use personalised support e.g. individualised comments or prompts to support tasks
- Give individualised attention
- Give positive praise and reinforcement
- Give general encouragement
- Reward effort not just success
- Give incentives for trying new skills
- Give learners options for presenting homework tasks using different media e.g. a mind map of a story, rather than an essay
- Use ICT to engage and sustain interest
- Develop positive peer reporting
- Gradually extend targets and expectations

Suitable for photocopying ©CIRCLE Collaboration all rights reserved

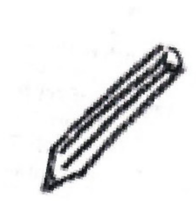

DEXTERITY & MANIPULATION (FINE MOTOR) SKILLS

SUPPORTS AND STRATEGIES

PLANNING PAGE

Learners Name

Class

Completed by

Position

Date

Summary of concerns from CPS:

Most successful strategies used:

Strategies to implement or use more consistently:

Plan (What, How, Who, When) :

Outcome:

Next steps:

CHILD INCLUSION RESEARCH INTO CURRICULUM LEARNING EDUCATION

Suitable for photocopying ©CIRCLE Collaboration all rights reserved

See www.thirdspace.scot/circle for more resources including interventions and practical supports. Copyright © 2020

Supplementary Material A7 Domains, sub-domains and examples of from CIRCLE Inclusive Classroom Scale (CICS)

Domain	Sub domain	Wording for of "exemplary" scoring within subdomain
Structures and routines	Decision making	Exemplary encouragement for learners to meaningfully participate in decisions
	Routines	Structured routines all exemplary, accommodates for individual preferences, high levels of consistency, promote sense of belonging
	Appeal of activities	Very positive perception and understanding of the value of activities offered to learners, variety evident, tailored to interests/cultures
	Expectations	Exceptionally clear expectations, communicated consistently by all adults in the school, and shared verbally, visually and through modeling
	Activity demands	Activities promote exceptionally appropriate and creative challenge and enjoyment with excellent engagement
Social environment	Empowerment	Excellent promotion of appropriate self-direction, proactive seeking of learners views/choices
	Provision of information	Proactive provision of information, several formats, shared and easily accessible
	Relationships	Exemplary, with diverse opportunities for positive social interaction and relationship building within class, school and wider community
	Support and facilitation	Expert support using variety of prompts / encouragement / demonstration, adapted to individual needs, and promotes independence over time
	Attitudes	Staff and peers in environment display, reinforce and value exceptional attitudes, and are highly empathic, non-judgmental
Physical environment	Availability of objects	Availability of objects exemplary, storage in close proximity, objects matched to characteristics/needs/cultures of learners, independently accessible, well maintained
	Visual supports	Excellent use of visual supports meeting individual needs, consistently used and applied
	Sensory space	Excellent sensory conditions, temperature pleasant, lighting and/ or noise levels optimised for sensory preferences
	Adequacy of Space	Exemplary availability of different areas and seating for meeting needs
	Accessibility of space	Accessibility exemplary, exceptional placing of furniture, barrier free

For full measure, see https://www.thirdspace.scot/circle/education-resources

Pages 19-29 "Primary school resource"

Pages 43-54 "Secondary school resource"

Supplementary Material A8 Circle Inclusive Classroom Scale (CICS) reflective question examples

Theme	Sub theme	Example reflective questions for teachers to support scoring
Structures and routines	Decision making	How do you support learners to be involved in decision-making? How do you ensure that everyone's voice is heard in the decision making process?
	Routines	How do you support learners to understand routines and to understand changes to routines? How do you prepare learners before each change?
	Appeal of activities	Are learners' interests and cultures utilised when designing lessons? Do good things just happen - are motivating activities timetabled across the week?
	Expectations	How do you agree expectations? How are expectations communicated to learners?
	Activity demands	Does work provide appropriate challenge and enjoyment for all? Is work differentiated to ensure achievable goals?
Social environment	Empowerment	How do staff assist learners in identifying and solving problems and building autonomy? How do staff encourage learners to express their needs, take responsibility or be autonomous?
	Provision of information	How is information shared with learners and parents/carers? Is information for learners and parents/carers easily available in a variety of formats?
	Relationships	How would you describe the relationship of (a) learners with staff, and (b) between the learners? Are activities regularly included to build relationships?
	Support and facilitation	Is the pace and quantity of information giving considered? Are complex instructions broken into clear steps?
	Attitudes	Is sensitivity used when asking learners to 'perform' in front of their peers e.g. reading aloud? Do adults model enthusiasm for tasks?
Physical environment	Availability of objects	Do learners have access to the equipment or resources they need? Are the available objects suited to the needs of learners consider seat and table height, types of pencils, pens, scissors etc?
	Visual supports	Is a consistent format for visuals used across the school? Are visual cues and supports in place?
	Sensory space	Is the environment adapted to meet sensory needs e.g. consider lighting and noise levels ? Can the sensory environment be altered easily?
	Adequacy of Space	Can you alter your classroom to suit the needs of learners? Is the class set up to promote good interaction?
	Accessibility of space	Can learners move around the classroom easily? Can learners access other areas of the school with ease?

For full measure, see https://www.thirdspace.scot/circle/education-resources

Pages 19-29 "Primary school resource"

Pages 43-54 "Secondary school resource"

Supplementary Material A9 Overview of the CIRCLE Participation Scale (CPS)

Domain	Description	Example items
The Environment	The physical environment refers to the physical layout of the classroom and the resources used within it.<br>The social environment concerns the attitudes, expectations and actions of peers and adults.	Sensory elements of classroom space have been considered for learner (e.g. noise, light, clutter)<br>Peers include learner in play/recreation activities
Structures and routines	Structures and routines are events that happen in the same way with regularity. The start, middle and end of the routine become predictable through repetition. Daily routines help learners to know and anticipate what comes next and social routines help them enjoy and interact with others.	Learner is aware of and adheres to normal school Routines<br>Learner is able to move between tasks, activities and/or classes during
Motivation	Motivation, including interest when engaging with activities, self-esteem, confidence, enthusiasm and pride.	Learner shows enthusiasm for activities at school<br>Learner shows pride in their achievements
Skills	Process skills (attention and concentration skills; organisation and planning skills)<br><br>Motor skills (posture and mobility skills; dexterity and manipulation skills)<br><br>Communication and Interaction skills (social, emotional and relationship skills; verbal and non-verbal communication skills)	Learner stays focused throughout an activity<br>Learner keeps themselves and their workspace organised<br>Learner is well co-ordinated when completing tasks<br>Learner can draw/write at a level appropriate for their developmental level<br>Learner verbalises their thoughts, ideas and questions appropriately

For full measure, see https://www.thirdspace.scot/circle/education-resources

Pages 33-39 “Primary school resource”

Pages 57-65 “Secondary school resource”
